# Protocol for Integrating Memory Workshops into the timely detection of Alzheimer’s Disease associated with prevention, awareness and action on risk factors

**DOI:** 10.1002/alz.088951

**Published:** 2025-01-09

**Authors:** Otelo Corrêa dos Santos Filho, Glaucia Dunley, Lucia França

**Affiliations:** ^1^ Human Aging Center, State University of Rio de Janeiro, Rio de Janeiro, Rio de Janeiro Brazil; ^2^ Human Aging Center ‐ State University of Rio de Janeiro, Rio de Janeiro, Rio de Janeiro Brazil; ^3^ Postgraduate Program in Psychology, Salgado de Oliveira University, Rio de Janeiro, Rio de Janeiro Brazil

## Abstract

**Background:**

The use of blood biomarkers predictive of the presence of amyloid Aβ and ApoE4 is an effective tool to facilitate a timely AD diagnosis. About a third of dementia cases can be attributed to potentially modifiable risk factors, and the incidence of the disease can be reduced by addressing these factors. Memory Workshops integrated with early detection and actions on risk factors is an operational strategy that is both therapeutic and preventive.

**Method:**

Memory Workshops and Timely Detection of Alzheimer’s Disease and Control of Risk Factors ‐ Memory Workshops will be held for elderly, with two components: Guided Autobiography (GAB), a life review technique, which provides the rescue, search and reinforcement of individual purposes, in a historical and sociocultural scenario common to the group, reviving and valuing the main aspects of the history of life aiming to develop new projects and the Popular Memory Collective based on psychoanalysis, which should address central themes such as significant losses, sexuality, melancholy, differentiating physiological needs, deficiencies, fears of death and illness. Elderly with cognitive complaints will be invited to undergo: 1 ‐ Collection of biomarkers for AD, with defined exclusion criteria. 2 – Recording and monitoring the evolution of risk factors for AD. Elderly people will also receive informative material about AD.

**Result:**

It is a protocol to be implemented from February 2024 in a partnership between the Human Aging Center of the State University of Rio de Janeiro and the Intergenerational State Secretariat for Youth and Human Aging of the State of Rio de Janeiro – Brazil – Memory workshops, timely detection strategies and management of risk factors will be carried out through appropriate training by health professionals from the Project 60+ Centers of the Secretariat of State in agreement with the State University of Rio de Janeiro. 100 Centers will be created in the State of Rio de Janeiro with an estimated care of 100 elderly people per Center. Training and coordination of the work will be carried out by the authors of this abstract

**Conclusion:**

It is a protocol to be implemented.

